# Fluid Resuscitation in Sepsis: Reexamining the Paradigm

**DOI:** 10.1155/2014/984082

**Published:** 2014-08-11

**Authors:** Poorna Madhusudan, Bharath Kumar Tirupakuzhi Vijayaraghavan, Matthew Edward Cove

**Affiliations:** ^1^Cardiothoracic Intensive Care Unit, NUHS, Singapore 119074; ^2^National University Hospital, 5 Lower Kent Ridge Road, Singapore 119074; ^3^Intensive Care Unit, NUHS, Singapore 119074

## Abstract

Sepsis results in widespread inflammatory responses altering homeostasis. Associated circulatory abnormalities (peripheral vasodilation, intravascular volume depletion, increased cellular metabolism, and myocardial depression) lead to an imbalance between oxygen delivery and demand, triggering end organ injury and failure. Fluid resuscitation is a key part of treatment, but there is little agreement on choice, amount, and end points for fluid resuscitation. Over the past few years, the safety of some fluid preparations has been questioned. Our paper highlights current concerns, reviews the science behind current practices, and aims to clarify some of the controversies surrounding fluid resuscitation in sepsis.

## 1. Introduction

The incidence of severe sepsis varies between 20 and 30% in most intensive care units and is a leading cause of mortality [1]. Fluid resuscitation is one of the cornerstones of management. Though there is a consensus on the need for adequate fluid therapy, the timing, type, and quantity of fluid resuscitation remain controversial. Furthermore, the optimal monitoring technique to guide fluid therapy continues to be debated; with mounting and sometimes contradicting evidence, the ideal fluid strategy is increasingly elusive.

Contemporary understanding of the pathophysiology of sepsis supports intensive fluid resuscitation in the initial phase. SIRS and sepsis incite widespread inflammatory responses at tissue and cellular levels altering homeostasis. Resultant circulatory abnormalities (peripheral vasodilation, intravascular volume depletion, increased cellular metabolism, and myocardial depression) lead to an imbalance between oxygen delivery and demand, worsening end organ injury and failure.

In a landmark paper, Rivers and colleagues demonstrated early goal directed therapy, targeting a specific central venous pressure (CVP) and mixed central venous oxygen saturation (ScVO_2_), improved mortality by 16% [2]. In response, the surviving sepsis guidelines recommend early aggressive fluid resuscitation during the “golden” hours [3]. Although adequate fluid resuscitation makes eminent physiological sense, the optimal amount, and type of fluid remain unclear. Our paper aims to clarify these issues by reviewing the latest evidence guiding these practices.

## 2. Monitoring Fluid Resuscitation

### 2.1. Static Monitors

In sepsis, it is important to identify which patients will respond to volume resuscitation. In the critically ill, this means identifying the patient whose cardiac output will improve with fluid administration, called preload responsiveness. Traditionally, static indicators such as CVP have guided therapy. However, historic and recent evidence suggest CVP is a poor predictor of fluid responsiveness. In a systematic review on the usefulness of the CVP, Marik et al. concluded that it is neither a good indicator of volume status nor a predictor of responsiveness to fluid therapy [4]. It has been suggested that CVP no longer be used to guide fluid therapy [5]; although it remains in the surviving sepsis guidelines, some authors suggest that these recommendations should be revisited [5]. In fact, recent evidence suggests CVP guided fluid resuscitation leads to venous congestion increasing the incidence of pulmonary complications in septic shock [6]. However, removal of CVP parameters from the guidelines may result in inadequate volume resuscitation and many centres continue to use static CVP measurement, despite evidence that it is an unhelpful guide for fluid administration. Furthermore, respiratory variation in the CVP is useful for predicting fluid responsiveness in spontaneously breathing patients [7].

Similarly, the pulmonary artery catheter (PAC) is unable to predict fluid responsiveness. Perhaps this is partly why the PAC is not associated with improved outcomes and its use has declined over the past two decades [8]. Although hemodynamic variables available from the PAC, such as the pulmonary capillary wedge pressure (PCWP), cardiac output (CO), and derived variables, are helpful for determining the type of circulatory shock and assessing response to therapy, none of these parameters predict preload responsiveness [9]. Furthermore, recent evidence casts doubt on the accuracy of hemodynamic data obtained from PACs [10].

### 2.2. Dynamic Monitoring

The most useful indicators of preload responsiveness are phasic changes in stroke volume and systolic blood pressure during positive pressure mechanical ventilation [11]. Stroke volume variation (SVV) is the ratio of maximal stroke volume difference during several respiratory cycles and the mean stroke volume over the same period [12]. Since the arterial pulse pressure depends on the amount of blood ejected during each systole (stroke volume), the pulse pressure variation covaries with SVV [13]. During positive pressure ventilation, inspiration increases the intrathoracic pressure reducing the right ventricular (RV) filling and right ventricular output if the RV is volume responsive. This causes the left ventricular filling and left ventricular (LV) output to decrease over successive beats if the LV is also volume responsive [12]. A SVV of >15% in patients receiving a tidal volume of >8 mL/kg or an SVV of >10% in patients receiving a tidal volume of 6 mL/kg accurately predicts preload responsiveness in patients with a closed chest [14–16].

Commercially available monitors such as the PiCCO, LiDCOplus, Volume View/EV1000, and the FloTrac use pulse contour analysis to indirectly determine the cardiac output and stroke volume variation. Pulse contour analysis is based on the relationship of the stroke volume, aortic compliance, and systemic vascular resistance [17]. Complex algorithms that account for reflection waves and aortic impedance are used to analyse the arterial wave and derive the stroke volume. The LiDCO uses pulse power analysis to convert the arterial waveform into a volume-time waveform which makes it less dependent on the shape of the pulse wave [18]. Although these devices are dependent on accurate calibration to measure CO, SVV and PPV are not dependent on calibration and, therefore, less affected by reliability concerns associated with these devices.

### 2.3. Indicators of Tissue Perfusion

The ultimate goal of fluid resuscitation is adequate tissue perfusion. However, dynamic monitoring does not measure tissue perfusion. Indicators of adequate perfusion include SVO_2_, ScVO_2_, and lactate. The surviving sepsis group recommends targeting ScVO_2_ of 70% within the first 6 hours of recognition of sepsis [19]. However, ScVO_2_ may be normal or even elevated in sepsis, for example, in patients with chronic liver disease. In contrast, hyperlactatemia is a more consistent finding in severe sepsis [20]. Normalization of lactate can be a useful target, alongside other hemodynamic parameters. Jansen et al. demonstrated reduced hospital mortality when targeting normalisation of lactate in a multicentre RCT [21].

A potentially useful measure of tissue perfusion is gastric mucosal pH. Since splanchnic circulation is compromised early during hypoperfusion, gastric blood flow is reduced. Changes in gastric mucosal pH (pHi), measured using a tonometer, reflect the adequacy of splanchnic perfusion [22]. The pHi is determined using a fluid or air filled balloon tipped nasogastric tube. The balloon contents equilibrate with the gases in the gastric lumen; therefore, changes in carbon dioxide (CO_2_) in the balloon reflect gastric luminal CO_2_. The pHi is calculated from the gastric lumen CO_2_ and blood bicarbonate; lower values indicate greater hypoperfusion. Although it is useful in prognosticating multiorgan failure and death in several conditions such as acute pancreatitis [23], trauma [24], and other critically ill patients [25], technical difficulties and potential sources of error in manual tonometer monitoring have prevented its widespread use [22]. Other tissue perfusion monitors such as Sidestream Dark Field imaging technique (SDF) [26], sublingual capnometry [27–29], and near infrared spectroscopy (NIRS) [30–33] have also been studied in critically ill patients. Although some studies have shown benefit, these monitors are not widely available and their clinical utility for delivery of bedside critical care remains to be established [33, 34].

## 3. Which Fluid?

Intravenous fluid therapy originated during the great cholera outbreak of the nineteenth century [35–38]. Fluids of various compositions were used, and studies tracing their composition indicate they resembled balanced crystalloids [39]. Balanced solutions are those with an electrolyte composition similar to that of plasma. However, the most commonly used crystalloid is 0.9% saline, which is not balanced. About 10 million litres of saline are used each year in the UK and 200 million litres are sold every year in the United States [40].

### 3.1. Crystalloids (Saline and Balanced Solutions)

0.9% saline is frequently referred to as “normal” saline. However, Awad and colleagues elegantly showed that this term entered medical practice based on colloquialism rather than sound physiological or scientific data [39]. There is certainly nothing normal about “normal” saline. The first documented use of “normal saline” was in the Lancet in 1888 [41]; however the solution described bore no resemblance to 0.9% saline. The widespread adoption of 0.9% saline was likely based on its isotonicity, as described in a single in vitro experiment on red cell lysis as well as the convenience and low cost of production [42].

Although there is no consensus on the superiority of balanced solutions over 0.9% saline, contemporary understanding of acid-base balance and recent observational evidence favours balanced solutions. The Stewart physicochemical approach [43] to acid-base dictates that infusion of large quantities of 0.9% saline will result in hyperchloremic acidosis. The strong ion difference (SID-sum of all the strong cations minus sum of all the strong anions) of plasma is maintained by the greater concentration of sodium relative to chloride in the plasma. Electroneutrality is maintained by anions such as bicarbonate (HCO_3_^−^), weak acids (HA), and hydroxyl ion (OH^−^). Decreases in the SID decrease the available “space” for these anions, ultimately reducing [OH^−^]. However, the dissociation of water (kw) must remain constant. Since kw is directly proportional to the product of [OH^−^] and hydrogen ion concentration [H^+^], decreases in [OH^−^] lead to increases in [H^+^] causing acidosis. Infusing 0.9% saline provides relatively more chloride, compared to sodium, resulting in a reduction in the strong ion difference which in turn lowers the pH, causing hyperchloremic acidosis [44].

Few studies have compared balanced crystalloids and 0.9% saline in patients with sepsis. However, there is substantial animal evidence that hyperchloremia causes harmful effects. In dogs, hyperchloremia causes progressive renal vasoconstriction and a fall in GFR in denervated kidneys [45]. In animal sepsis models, infusions of 0.9% saline increase inflammatory cytokines, worsen hypotension and hyperlactatemia, are more likely to cause renal failure, and ultimately increase mortality [46–48]. In patients without sepsis undergoing aortic aneurysm, repair use of 0.9% saline (NS), when compared to lactated ringer's (LR), was associated with increased platelet use (223 mL LR group versus 392 mL in NS group, median difference −169, 95% confidence interval (CI) −814 to −13) and increased interventions for acidosis (volume of bicarbonate used for acidosis; 40.2 ± 64.0 mL NS group versus 3.8 ± 15.5 mL LR group) [49]. In a randomized double-blind comparison of lactated Ringer's solution and 0.9% saline during renal transplantation, lactated Ringer's was associated with less hyperkalaemia and acidosis [50]. In a recent large observation study, Shaw et al. compared adult patients undergoing major open abdominal surgery receiving either 0.9% saline (30,994 patients) or a balanced crystalloid solution (PlasmaLyte) (926 patients). Unadjusted hospital mortality was higher in the saline group (5.6% versus 2.9%; *P* < 0.001). After propensity score matching, patients receiving 0.9% saline received more fluid (1976 mL [± 1560] versus 1658 mL [± 1288], *P* < 0.001), more buffer orders (6.3% [95% CI 5.5–7.3] versus 4.2% [95% CI 3.1–5.7], *P* = 0.02), and more transfusions (11.5% [95% CI 10.3–12.7] versus 1.8% [95% CI 1.2–2.9], *P* < 0.001) and the use of dialysis was nearly fivefold greater (1.0% [95% CI 0.05–1.8] versus 4.8% [95% CI 4.1–5.7], *P* < 0.001) when compared to PlasmaLyte [51].

In Sub-Saharan African children with severe sepsis, fluid boluses with saline or 5% Albumin in saline result in increased mortality when compared to no fluid boluses [52]. Although the predominance of malarial sepsis could lead to severe anaemia following fluid boluses, theoretically contributing to this observation, it is intriguing to postulate that, in the absence of modern ICU interventions, the negative effects of 0.9% saline may be magnified. Furthermore, post hoc analysis suggests cardiovascular collapse accounted for the excess mortality in the fluid bolus groups [53]. There is clearly mounting evidence suggesting that hyperchloremia associated with use of 0.9% saline does have significant clinical implications, cannot be ignored and should at least give pause to continued saline only resuscitation in sepsis.  Table 1 summarizes the studies examining crystalloids.

### 3.2. Colloids

There are fundamental differences between crystalloids and colloids. Crystalloids are predominantly based on sterile water to which electrolytes have been added. Colloids have an additional “colloidal” component that does not freely diffuse across semipermeable membranes, in theory making them more effective volume expanders. Colloids are the preferred resuscitation fluids in Europe and Australasia [54]. Albumin, hydroxyethyl starch (HES), and gelatins are the three classes of colloid commonly used.

However, the safety profile of certain colloids in patients with sepsis has recently been challenged. In fact, safety concerns have existed since their introduction. Scheirhout and colleagues, in a meta-analysis of 37 RCTs in critically ill patients, found that resuscitation with colloids (albumins, gelatins, dextrans, and starches) increased risk of mortality by 4% (95% CI 0–8%) [55]. A separate French multicentre study found gelatins [odds ratio (OR) 4.81 (95% CI 2.01–11.51 *P* = 0.0005)] and dextrans [OR 3.83 (95% CI 1.17–12.60 *P* = 0.02)] were independent risk factors for anaphylactoid reactions [56]. Furthermore, Dextran 70 has been shown to decrease Factor VIII procoagulant activity, factor VIII related antigen, and ristocetin cofactor activity [57] resulting in coagulopathies.

Based on adverse outcome reports, such as renal dysfunction and coagulopathy, high molecular weight starches have already been phased out in favour of HES (130/0.42). The adverse effects of HES were considered benign, transitory, dose dependent, and related to only high molecular weight starches. However, HES (130/0.42) is not readily excreted and there is evidence it accumulates in the skin, liver, kidney, and reticuloendothelial system [58]. It is suggested that the lower degree of substitution and lower molecular weight of HES (130/0.42) facilitate greater uptake in the tubular epithelium leading to osmotic nephrosis and requirement of renal replacement therapy and, therefore, could be more harmful than its predecessors [58–60].

In the recent Crystalloid versus Hydroxyethyl starch Trial (CHEST), the effect of fluid resuscitation with HES (130/0.4) was compared with 0.9% saline among 7000 patients admitted to an intensive care unit [61]. The study found no difference in 90 day mortality between the groups; however, patients receiving HES required renal replacement therapy more frequently (RR 1.21, 95% CI 1.00–1.45, *P* = 0.04). The study also demonstrated more adverse events with the use of HES.

A second trial recently randomized 804 patients with severe sepsis to receive either HES (130/0.42) or Ringer's acetate, the 6S trial [62]. The primary outcome of death or dialysis dependence at 90 days occurred in 51% of the HES group compared to 43% in the Ringer's group (*P* = 0.03) with more patients in the HES group receiving renal replacement therapy (22% versus 16%, *P* = 0.04). Since Ringer's acetate was the carrier of HES in the intervention arm, it enabled the investigators to examine only the effects of HES between the two groups. It is conceivable that CHEST study did not identify a mortality difference due to harmful effects exerted by 0.9% saline in the control arm. In addition, a recent meta-analysis by Zarychanski et al. concluded that HES was associated with significant increase in mortality and acute kidney injury in patients with sepsis [63].

However, some studies contradict these findings, suggesting benefit to HES use. The CRYSTMAS trial was a prospective multicentre, double blind randomized study comparing the hemodynamic efficacy and safety of HES (130/0.4) with 0.9% saline in severe sepsis. The authors found significantly less volume of HES was required to achieve hemodynamic stability (1379 ± 886 mL in HES versus 1709 ± 1164 mL in saline group, *P* = 0.0185) and found no difference in the rate of AKI or RRT [64]. Unfortunately, it lacked power to address renal safety, and based on current evidence, the FDA has issued a boxed warning for HES. It would appear the risks associated with HES use in sepsis outweigh any volume expansion benefits and its use in sepsis cannot currently be recommended. Given the concerns with synthetic colloids, albumin has reemerged as a good alternative. The recent surviving sepsis campaign guidelines advocate the use of albumin for volume expansion after the use of crystalloids [19].

Apart from the hemodynamic efficacy that albumin confers, it is reported to have antioxidant and anti-inflammatory activity [65]. The postulated mechanisms include an increase in plasma thiol levels, modulation of cytokine activity, binding of endotoxin, and protection of glycocalyx. It also alters drug binding and reduces nitric oxide, attenuating vasodilatation [66].

The SAFE study compared albumin and saline resuscitation in 6997 patients [67]. Twenty-eight-day mortality was no different between both groups (726 albumin group versus 729 0.9% saline group, *P* = 0.87). The study concluded that albumin and 0.9% saline are clinically equivalent for fluid resuscitation in the ICU. However, post hoc analysis of the sepsis subgroup indicates that resuscitation with albumin may reduce the mortality in patients with severe sepsis, confirming possible additional protective mechanisms conferred by albumin. Furthermore, a large meta-analysis showing resuscitation with albumin solutions in sepsis was associated with lower mortality [68]. Although many of the studies included had not used proper methodology, the results suggest that albumin does not have specific adverse effects in sepsis.  Table 2 summarizes studies examining colloids.

A separate multicentre study in Italy, the ALBIOS trial, recruited 1800 patients with sepsis or septic shock and compared resuscitation with 20% albumin or a crystalloid the results of which are not yet published. Similarly, another large trial involving 800 patients with sepsis in France resuscitated either with 20% albumin or normal saline, conducted by the EARSS study group, is yet to publish its results. Together, the results of these two large trials will help confirm whether albumin has additional protective effects.

The recent CRISTAL randomized trial examined the effects of fluid resuscitation with colloids versus crystalloids on mortality in critically ill patients with hypovolemic shock [69]. It was a multicentre, open label randomized clinical trial stratified by case mix (sepsis, trauma, or hypovolemic shock without sepsis or trauma). They used colloids (*n* = 1414; gelatins, dextrans, hydroxyethyl starches, 4% or 20% of albumin) or crystalloids (*n* = 1443; isotonic saline, hypertonic saline, or Ringers lactate) for fluid interventions, other than fluid maintenance throughout the ICU stay. There was no difference in 28-day mortality between the two groups (359 in colloid group versus 390 in crystalloid group, *P* = 0.26). However, a secondary outcome, 90-day mortality, was lower in patients receiving colloids, but it is difficult to draw strong conclusions from this study due to the heterogeneity of fluid composition in the two groups.

From the many trials that have been carried out so far, it is clear that some synthetic colloids should be avoided in sepsis and that 0.9% saline may have disadvantages over balanced crystalloids. However, whether to select albumin over a crystalloid remains uncertain. Based on the SAFE study, one potential advantage of albumin is that less fluid is ultimately required to achieve hemodynamic end goals. This will only prove beneficial, if a more positive balance is associated with worse outcomes.

## 4. How Much Fluid?

**Figure 1:**
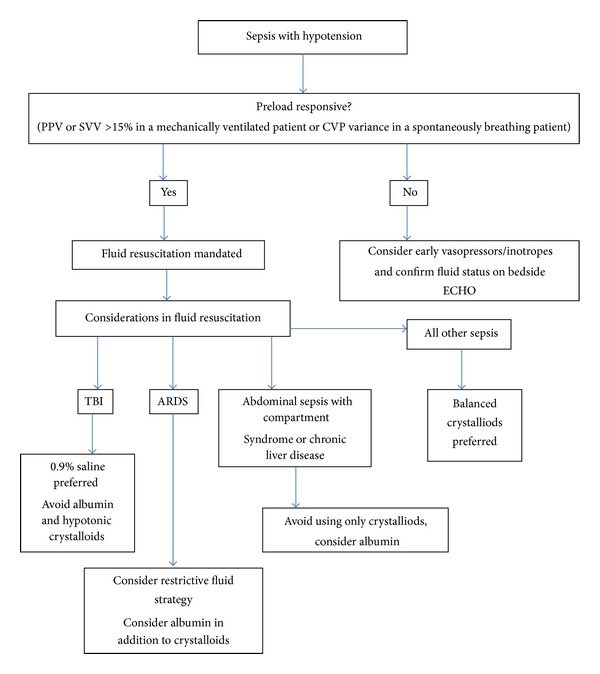
Algorithm to guide fluid therapy in the septic patient.

Boyd and colleagues retrospectively reviewed the association of positive fluid balance at 12 hours and at 4 days in 778 patients of the Vasopressin in Septic Shock (VASST) study [70]. They found that the quartile that had the least positive balance at 12 hours [0.569 (0.405–0.799) for Quartile 1 and 0.581 (0.414–0.816) for Quartile 2] and at 4 days [0.466 (0.299–0.724) for Quartile 1 and 0.512 (0.339–0.775) for Quartile 2] had a lower hazard ratio relative to the quartile with the maximum positive balance. Furthermore we know that a fluid restrictive strategy is beneficial in patients with concomitant ARDS [71]. Although it is expected that 3 to 4 times the volume of crystalloids may be required to achieve the hemodynamic efficacy of colloids, the SAFE study found that the volume of saline used was only 40% more than albumin, perhaps because the clearance of crystalloids is decreased during the stress response of critical illness. Furthermore, there are settings where one fluid is clearly advantageous, such as sepsis patients with traumatic brain injury where albumin and hypotonic resuscitation fluids should be avoided [72]. Similarly, patients requiring fluid restrictive strategy, such as those with ARDS or concomitant abdominal compartment syndrome, might benefit from an albumin based strategy (Figure 1).

## 5. Conclusion

In conclusion, a perfect one-size-fits-all fluid strategy does not exist. In sepsis, clinicians should understand the limitations and potential benefits of each strategy. Each fluid should be considered a drug, with specific pharmacokinetic, pharmacodynamic, and adverse effect profiles, which can be carefully matched to the patient. Whichever fluid is chosen, resuscitation should be titrated to evidence based targets, combining clinical assessment, such as signs of tissue perfusion with dynamic hemodynamic monitoring. Balanced crystalloids may be preferred first choice, followed by albumin, based on their comparative safety profiles. 0.9% saline should only be used after consideration of its potential to cause harm and current evidence would suggest starches (HES) should be avoided in sepsis.

## Figures and Tables

**Table 1:** Summary of studies evaluating crystalloids.

Author	Year	Study design	Sample size	Study fluid	Primary endpoint	Comments
Wilcox [45]	1983	Animal experiment	48	Chloride rich solutions	Regulation of renal blood flow	Increased renal vasoconstriction and ↓GFR^a^ with chloride rich solutions

Waters et al. [49]	2001	Prospective randomized study	66	0.9% Saline versus lactated Ringer	Multiple outcomes studied	Increased use of blood products and acidosis with 0.9% Saline

O'Malley et al. [50]	2005	Randomised clinical trial	51	0.9% Saline versus lactated Ringer	Creatinine concentration on POD3^b^	No difference; but Ringer's was associated with less hyperkalemia and acidosis

Shaw et al. [51]	2012	Observational	31,920	0.9% Saline versus balanced crystalloid	Major morbidity	Higher mortality, increased transfusion requirements, dialysis requirements, and increased buffer requirements in saline group

Maitland et al. [52]	2011	Multicentric randomized trial	3141	Albumin bolus and saline bolus	Mortality	Boluses resulted in increased mortality

Table summarizing studies evaluating crystalloids. ^a^Glomerular Filtration Rate, ^b^Postoperative Day 3.

**Table 2:** Summary of studies evaluating colloids.

Author	Year	Study design	Sample size	Study fluid	Primary endpoint	Comments
Schierhout and Roberts [55]	1998	Meta-analysis	1315	All colloids	Mortality	Increased mortality

Laxenaire et al. [56]	1994	Multicentre prospective	19593	All colloids	Adverse effects	Gelatins and dextrans-independent risk for anaphylactoid reactions

Myburgh et al. [61]	2012	RCT^a^	7000	HES^b^ versus 0.9% saline	90-day mortality	HES^b^ associated with increased incidence of RRT

Perner et al. [62]	2012	RCT^a^	804	HES^b^ versus Ringer's acetate	Death/dialysis dependence at 90 days	Death and dialysis dependence more in HES

Zarychanski et al. [63]	2013	Meta-analysis	10,290	HES^b^	Mortality and AKI^c^	Significant increase in risk of mortality and AKI^c^

Guidet et al. [64]	2012	RCT^a^	196	HES^b^ versus 0.9% saline	Hemodynamic efficacy and safety	HES^b^ better hemodynamic efficacy and no difference in AKI^c^

Finfer et al. [67]	2004	RCT^a^	6997	Albumin versus 0.9% saline	28-day mortality	No difference

Myburgh et al. [72]	2007	Post hoc analysis of SAFE trial	460	Albumin versus 0.9% saline	Safety in TBI^d^	Albumin unsafe for TBI^d^

Delaney et al. [68]	2011	Meta-analysis	1977	Albumin	Safety for resuscitation	Albumin associated with lower mortality

Annane et al. [69]	2013	RCT^a^	2857	Colloids versus crystalloids	28-day mortality	No difference

Table summarizing studies evaluating colloids. ^a^Randomized Controlled Trial, ^b^Hydroxyethyl starch, ^c^Acute Kidney Injury, ^d^Traumatic Brain Injury.
